# Supplementary material for: Impact of blindness onset on the representation of sound categories in occipital and temporal cortices
Source: eLife. 2022 Sep 7;11:e79370. doi: 10.7554/eLife.79370 (PMC9451537; doi:10.7554/eLife.79370)
Supplement: Supplementary file 2. [file elife-79370-supp2.docx]

**Table 2. Categories and stimuli.**

| CATEGORIES | STIMULI |
| --- | --- |
| BIRDS | Canary  Owl  Seagull |
| MAMMALS | Dog  Donkey  Horse |
| HUMAN  VOCALIZATIONS | “*Fro”-* Woman  “*BaBe”* - Man  “OOO”-Man |
| HUMAN  NON VOCALIZATIONS | Women laughing  Man crying  Women yawning |
| TOOLS | Hairdryer  Saw  Toothbrush |
| GRASPABLE  OBJECTS | Guitar  Keyboard  Telephone |
| BIG MECHANICAL  OBJECTS | Church-bell  Traffic  Train |
| ENVIRONMENTAL  SCENES | Storm  River  Wind |
