# Supplementary material for: Impact of blindness onset on the representation of sound categories in occipital and temporal cortices
Source: eLife. 2022 Sep 7;11:e79370. doi: 10.7554/eLife.79370 (PMC9451537; doi:10.7554/eLife.79370)
Supplement: Supplementary file 3. [file elife-79370-supp3.docx]

**Table SI 5. Groups’ mean beta values for each ROI and for every category.**

|  | **OCC ROI [all Sounds EB>SC]** | | | | **TEMP ROI [all Sounds SC>EB]** | | | |
| --- | --- | --- | --- | --- | --- | --- | --- | --- |
| *Categories* | *Animal* | *Human* | *Manip.* | *Big&Place* | *Animal* | *Human* | *Manip.* | *Big&Place* |
| **SC** | 0.47 | -0.16 | 0.14 | 0.11 | 23.26 | 32.96 | 27.50 | 26.06 |
| **EB** | 5.01 | 4.46 | 6.02 | 5.02 | 15.14 | 18.74 | 16.21 | 15.54 |
|  | **OCC ROI [all Sounds LB>SC]** | | | | **TEMP ROI [all Sounds SC>LB]** | | | |
| *Categories* | *Animal* | *Human* | *Manip.* | *Big&Place* | *Animal* | *Human* | *Manip.* | *Big&Place* |
| **SC** | -0.22 | 0.32 | 0.95 | 0.62 | 14.50 | 18.36 | 15.39 | 14.28 |
| **LB** | 10.95 | 10.62 | 10.62 | 9.81 | 9.78 | 11.93 | 9.58 | 8.89 |
