# Supplementary material for: Impact of blindness onset on the representation of sound categories in occipital and temporal cortices
Source: eLife. 2022 Sep 7;11:e79370. doi: 10.7554/eLife.79370 (PMC9451537; doi:10.7554/eLife.79370)
Supplement: Supplementary file 4. [file elife-79370-supp4.docx]

**Table SI 3. R and p values (FDR corrected for 8 comparisons) from RSA correlation between the Occipital DSMs & representational models**

|  | **GROUPS** | | | | | | | |
| --- | --- | --- | --- | --- | --- | --- | --- | --- |
|  | *ROI: EB>SC (from univariate)* | | | | *ROI: LB>SC (from univariate)* | | | |
| **MODELS** | SC | | EB | | SC | | LB | |
|  | *r* | *p* | *r* | *p* | *r* | *p* | *r* | *p* |
| Behavioural | 0.06 | *N.S.* | ­–0.08 | *N.S* | 0.0002 | *N.S* | –0.055 | *N.S* |
| Human | 0.04 | *0.26* | **0.20** | ***.0012*** | –0.03 | *N.S* | **0.16** | ***.014*** |
| Animal | –0.09 | *N.S* | -0.08 | *N.S* | –0.07 | *N.S* | –0.099 | *N.S* |
| Manipulable | –0.08 | *N.S* | -0.04 | *N.S* | –0.08 | *N.S* | –0.06 | *N.S* |
| Big & Places | –0.07 | *N.S* | -0.04 | *N.S* | –0.02 | *N.S* | –0.03 | *N.S* |
| HNR | –0.03 | *N.S* | -0.04 | *N.S* | –0.02 | *N.S* | 0.03 | *N.S* |
| Pitch | 0.04 | *N.S* | 0.0050 | *N.S* | –0.004 | *N.S* | 0.004 | *N.S* |
