# Supplementary material for: Impact of blindness onset on the representation of sound categories in occipital and temporal cortices
Source: eLife. 2022 Sep 7;11:e79370. doi: 10.7554/eLife.79370 (PMC9451537; doi:10.7554/eLife.79370)
Supplement: Supplementary file 5. [file elife-79370-supp5.docx]

**R and p (FDR corrected for 6 comparisons) values from RSA correlation between Temporal DSMs & representational models**

|  | **GROUPS** | | | | | | | |
| --- | --- | --- | --- | --- | --- | --- | --- | --- |
|  | *TEMP ROI: SC>EB (from univ.)* | | | | *OCC ROI: SC>LB (from univ.)* | | | |
| **MODELS** | SC | | EB | | SC | | LB | |
|  | *r* | *p* | *r* | *p* | *r* | *p* | *r* | *p* |
| Behavioral | **0.18** | ***p<0.001*** | **0.13** | ***p<0.001*** | **0.22** | ***p<0.001*** | **0.16** | ***p<0.001*** |
| Human | **0.24** | ***p<0.001*** | **0.14** | ***p<0.001*** | **0.26** | ***p<0.001*** | **0.18** | ***p<0.001*** |
| Animal | –0.04 | *N.S* | –0.13 | *N.S* | **–**0.04 | *N.S* | –0.22 | *N.S* |
| Manipulable | –0.15 | *N.S* | –0.14 | *N.S* | –0.15 | *N.S* | –0.19 | *N.S* |
| Big & Places | –0.01 | *N.S* | 0.03 | *N.S* | –0.01 | *N.S* | –0.06 | *N.S* |
| HNR | –0.07 | *N.S* | –0.08 | *N.S* | –0.19 | *N.S* | –0.14 | *N.S* |
| Pitch | 0.03 | *N.S* | 0.05 | *N.S* | **0.13** | ***p=0.006*** | 0.08 | ***p=0.03*** |
